# MfMRI assessment of muscle activation by swallowing exercises in laryngectomized individuals: investigating conventional and resistance-based exercises using the swallow exercise aid (SEA)

**DOI:** 10.1007/s00405-025-09727-9

**Published:** 2025-11-05

**Authors:** Marise Neijman, Leon C. ter Beek, Loes M.M. Braun, Frans J.M. Hilgers, Martijn M. Stuiver, Lisette van der Molen, Michiel W.M. van den Brekel, Maarten J.A van Alphen

**Affiliations:** 1 Department of Head and Neck Oncology and Surgery, the Netherlands Cancer Institute, Plesmanlaan 121, 1066CX Amsterdam, the Netherlands; 2 Amsterdam Center for Language and Communication (ACLC), University of Amsterdam (UvA), Binnengasthuisstraat 9, 1012ZA Amsterdam, the Netherlands; 3 Department of Medical Physics, the Netherlands Cancer Institute, Plesmanlaan 121, 1066CX Amsterdam, the Netherlands; 4 Department of Radiology, the Netherlands Cancer Institute, Plesmanlaan 121, 1066CX Amsterdam, the Netherlands; 5 Center for Quality of Life and Division of Psychosocial Research and Epidemiology, the Netherlands Cancer Institute, Plesmanlaan 121, 1066CX Amsterdam, the Netherlands

**Keywords:** Total laryngectomy, Swallowing, Strength exercises, Muscle activation, MR imaging, T2 mapping

## Abstract

**Purpose:**

After total laryngectomy, dysphagia is common due to changes in anatomy and physiology. Currently, it is unclear if the remaining muscles still can be activated with swallowing exercises. This study aims to assess which muscles, if any, are activated after conventional or resistance-based exercises with the Swallowing Exercise Aid (SEA) using mfMRI-based T2maps.

**Methods:**

Five laryngectomized individuals underwent mfMRI before and after six exercises. Three conventional (Effortful Swallow, Masako, Shaker) and three SEA exercises (Chin Tuck, Jaw Opening and Effortful Swallow Against Resistance (CTAR, JOAR and ESAR, resp.)) were assessed. T2 mapping scans were made pre and post all exercises carried out until exhaustion. Muscles of interest were the medial and lateral pterygoids, intrinsic and extrinsic tongue, suprahyoid, superior pharyngeal constrictor, masseter and sternocleidomastoid muscles. Primary parameter was the difference (Δ) of the post- and pre-exercise T2 map values in milliseconds.

**Results:**

In total, 1.490 annotated muscles were analyzed. Following the swallowing exercises, true positive Δ T2 map values were found in up to 90% of these muscles. Each exercise (conventional and SEA-based) activated at least three muscles of interest, in varying numbers per exercise. Among all annotated muscles, percentages of positive Δ T2 map values were highest for the lateral pterygoid muscles following the SEA exercises CTAR and JOAR (70 and 90%, resp.).

**Conclusion:**

This mfMRI study shows that after total laryngectomy swallowing muscles still can be activated with swallowing exercises. The SEA-based exercises CTAR and JOAR showed the highest percentage of bilateral activation.

**Supplementary Information:**

The online version contains supplementary material available at 10.1007/s00405-025-09727-9.

## Introduction

Total laryngectomy is the treatment of choice for primary advanced laryngeal or hypopharyngeal cancer, for salvage surgery in cases of recurrence, or for resolving severe functional or aspiration problems persisting after organ-preservation treatment [1]. The standard laryngectomy procedure involves removal of the entire larynx, hyoid bone, and infrahyoid musculature. As a result, the upper and lower airways are disconnected, leading to a tracheostoma in the neck. A (neo)pharynx is reconstructed through primary mucosal closure (T/Y shape, vertical or horizontal). The transected suprahyoid muscles are usually reattached to the superior constrictor pharyngeal muscles, and often, the cricopharyngeal muscles are myotomized.

These anatomical changes lead to physical and biomechanical alterations not only in breathing, speaking, and olfaction, but also in eating and drinking. The anatomy and the inherent physiology changes can cause altered bolus transit times, reduced swallowing efficiency, impaired pharyngeal clearance, neopharynx stenosis, weak tongue base pressure, and loss of muscular coordination [2]. The literature presents varying statistics on the incidence and prevalence of swallowing issues post-laryngectomy. Maclean et al. (2009) found that even up to 72% of laryngectomized patients experience long-term self-reported swallowing problems [3]. 

To improve bolus transit times during eating and drinking and diminish residue, patients may benefit from increased pressure, which depends on the strength of the involved muscles. There are several conventional muscle-strengthening (swallowing) exercises, such as the Effortful Swallow (cES), the Masako, and the Shaker [4–8]. Although, these conventional exercises do not meet the principles of muscle-strength training (e.g., training at 60–80% of the 1 Repetition Maximum, increasing the resistance and repetitions) [9], they can to some extent restore swallowing function in participants with oropharyngeal dysphagia, but they have never been thoroughly evaluated in laryngectomized patients.

However, some (swallowing) exercises that adhere to the principles of muscle strength training (e.g., the Chin Tuck Against Resistance (CTAR), Jaw Opening Against Resistance (JOAR), and Effortful Swallow Against Resistance (ESAR) against resistance, all performed with the Swallowing Exercise Aid (SEA) device) exist. These resistance-based exercises have been evaluated in this specific patient population [10]. After six weeks of training, a significant increase in muscle strength, swallowing capacity, and swallowing efficiency was observed. Furthermore, quality of life improved, oral intake increased, and dysphagia symptoms decreased. These findings raise the question of whether the transected and reunited muscles of the pharynx can still be activated, or if other muscles compensate for the altered anatomy and physiology in laryngectomized individuals. Therefore, the present study aims to assess which muscles after total laryngectomy are activated with conventional swallowing exercises and swallowing exercises performed against resistance with the SEA, using mfMRI based T2 mapping.

## Methods

The Medical Ethical Committee approved the study protocol (METC21.0906/N21SEM). The guidelines of the Helsinki Declaration were followed, and written informed consent was obtained from each participant before inclusion.

### Participants

Between July and October 2022, laryngectomized individuals were recruited through the Department of Head and Neck Oncology and Surgery of the Netherlands Cancer Institute and via the Dutch Patient Association for Head and Neck Cancer (PVHH). Participants had to be in complete remission, at least six months post-surgery and, if relevant, six months after postoperative (chemo) radiotherapy. To establish a more homogeneous study population, only participants with a primary T/Y shape closure of the neopharynx were considered for inclusion.

Five participants were included and signed informed consent. All participants were male. The median age was 62 years (range 54–67), and the median time after laryngectomy was 49 months (range 9–192), see Table 1. Three out of five participants had self-reported swallowing problems. Most reported complaints were inefficient swallowing (residue), the need for compensation techniques and prolonged time needed for eating and drinking.

**Table 1:** Participant characteristics

Participant				Tumor		Total laryngectomy			Radiotherapy		
No.	Sex	Age	BMI	Location	TNM	Year	Indication	Time since*	Yes/no	Dose (Gray)	Time since*
P1	M	66	39.4	Larynx	pT4aN0Mx	2021	Curative	9	Yes	56	6
P2	M	54	25.2	Larynx	pT4N2aM0	2021	Curative	16	Yes	56	14
P3	M	61	28.0	Larynx	cT4aN0M0	2018	Curative	49	No	-	-
P4	M	62	27.7	Larynx	pT4N1M0	2006	Curative	192	Yes	56	189
P5	M	67	25.5	Larynx	T3N0M0	1998	Functional	104	Yes	Unknown	290

*BMI* Body Mass Index, *time in months

### Swallowing exercises

To investigate how the altered musculature reacts to exercises for swallowing after laryngectomy, six different exercises (of which three conventional and three resistance-based performed with the SEA) were investigated. Participants were asked to perform the exercises at least 30 times and then continue until exhaustion to ensure that any muscle activation would be detectable with the mfMRI. The first-generation plastic SEA was used due to its MRI compatibility and safety [11]. 

### Conventional swallowing exercises

The conventional swallowing exercises included the cES, Masako and Shaker exercises [12–14]. For the cES exercise, the participant was instructed to swallow as effortful as possible. For the Masako exercise, the participant was asked to hold the tongue between the teeth while swallowing. For the Shaker exercise, the participant was instructed to lie on their back and repetitively lift the head to look at the toes, keeping the shoulders flat against the bench of the MRI.

### Swallow exercise aid

**Fig. 1:**
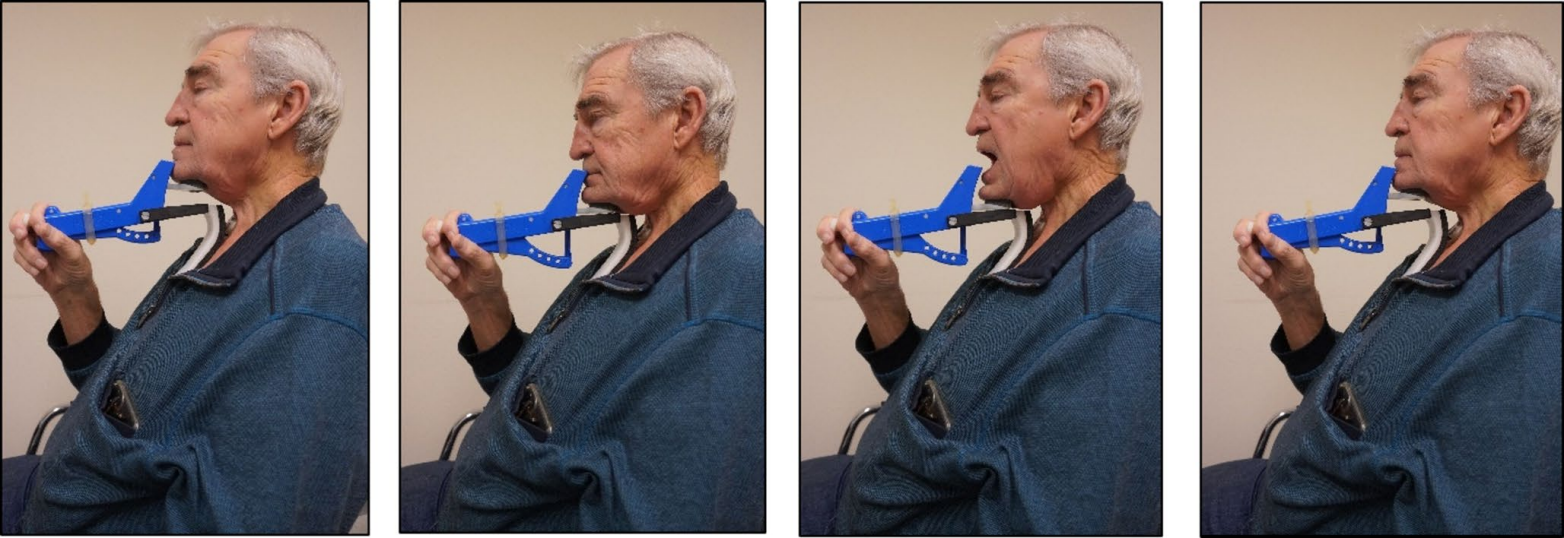
Exercises performed with the Swallowing Exercise Aid (SEA). From left to right: (1) Resting position, (2) Chin Tuck against Resistance (CTAR), (3) Jaw Opening against Resistance (JOAR), and (4) Effortful Swallow against Resistance (ESAR). The (adjustable) position of the silicon (Active) band around the handle (right in front of the hand) determines the increasable resistance against which exercise has to be performed

In Fig. 1, the SEA exercises are displayed. For the CTAR exercise, the participant was instructed to press their chin towards their chest until the chin bar clicked onto the chest bar, providing audible and tactile feedback. For the JOAR exercise, the participant was asked to press the mandible downward while simultaneously opening their mouth to bring the chin bar into contact with the chest bar. For the ESAR exercise the participant was instructed to press the chin bar downward to 50% of its full range and maintain that position. Simultaneously, the participant was instructed to perform a forceful swallow with their mouth closed while keeping the chin bar at the 50% position.

### Procedure

Before the mfMRI evaluation, the participant received instructions regarding the exercises and study protocol. The predetermined order of exercises was cES, Masako, Shaker, CTAR, JOAR, and ESAR. This order was chosen because SEA exercises might exhaust participants more due to the resistance. When a participant was unsure about completing all exercises due to fatigue, they were asked to start with the SEA exercises because an underlying aim of this study was to support or explain the promising results seen in the phase II trial [9]. 

Progressive resistance levels for SEA exercises for each participant were determined using silicon ActiveBands during a trial session. During that session, the participants completed ten repetitions of each SEA exercise at the highest resistance level with one silicon band. If they felt unable to achieve 30 repetitions, the resistance was decreased until they found the right level for each exercise. Subsequently, the participant was instructed to lie supine with the dStream head-spine detection coil securely affixed to the examination table to optimize the signal-to-noise ratio.

Once the participant was positioned inside the MRI, the survey and pre-exercise T2 map scans were conducted. Then, the participant was carefully moved out of the MRI and given instructions to sit upright (or stay in supine position in case of the Shaker exercise). In the presence of a Speech and Language Pathologist (SLP), the participant performed one of the six swallowing exercises in the predetermined order until reaching the point of exhaustion, with a minimum of 30 repetitions. The SLP monitored and recorded the number of repetitions and the exercise duration. When the participant reached the point of exhaustion (indicating an inability to perform more repetitions of that specific exercise), the participant was instructed to return to a supine position and were immediately repositioned inside the MRI. Then, the survey and post-exercise T2 map scans were acquired.

To prevent any potential interference from carry-over effects of the prior exercise on the subsequent one, participants had to rest at least 10 min between the exercise cycles after the post-exercise scan. Following each post-exercise T2 map scan, the participant was slid out of the MRI. During the resting period, the participant performed different activities, outlined in a study-specific lymph drainage protocol that was created in collaboration with a specialized edema therapist (see Appendix 1). The purpose of this lymph drainage protocol was to activate and support lymph drainage in the head and neck area to restore the water ratio to baseline in the activated muscles before proceeding to the next pre-exercise T2 map mfMRI scan. After the resting period, another pre-exercise T2 map MRI was made, followed by the subsequent exercise, another set of post-exercise survey and T2 map scans, and a new resting period. This cycle was repeated until the participant had completed all six exercises or had decided to stop.

#### MRI acquisition

The mfMRI technique with its derived T2 maps is a non-invasive and effective method for visualizing muscle activation. This technique enables detection of changes in water distribution within muscles, indicated by an increase in the transverse relaxation time constant [15]. This T2 spin-spin relaxation time constant, also known as the T2 value, measured in milliseconds (ms), is a parameter characterizing the decay of the MR signal in the transverse plane, and therefore represents the duration of transverse magnetization of water protons within a voxel. Pre- and post-exercise T2 map scans allow for the assessment of changes in the T2 values of muscle tissue. This validated method provides valuable insights into muscle activation dynamics [16, 17]. 

All scans were acquired on a 3 T Ingenia scanner from Philips Healthcare (Best, the Netherlands) with a dStream head-spine coil. For the measurement of T2 values, the same settings and software outlined in our earlier work were employed [11]. Scans were made in the axial plane. T2 values were determined for specific muscles by generating a T2 map. This quantitative map provides spatial distributions of T2 values linked to anatomical structures within the head and neck region.

A rapid T2 sequence was needed to analyze the exercises’ potential effects before muscle tissue recovery. Therefore, the specialized k-t-T2 accelerated research software patch from Philips Healthcare was applied. Each survey scan lasted 30 s, and every T2 map scan had an approximate duration of 4 min.

The multi-slice multi-echo T2 weighted turbo spin-echo (SE) sequence had a field of view measuring 170 mm in the anterior-posterior and right-left directions and 129 mm in the foot-head direction. Voxel size during acquisition was 1.2 × 1.2 mm in plane, with a reconstructed voxel size of 0.6 × 0.6 mm, using contiguous slices, each 3 mm thick. The sequence incorporated 12 echo times ranging from 16ms to 104ms, with 8ms increments. The repetition time was set at 4211ms. Half scan was 0.613, and the sense acceleration factor was 2 in the left-right direction. A voxel wise fitting procedure was applied to the T2 decay curve using these 12 echo times, generating a quantitative 3D T2 map across the entire field of view.

### Registration and annotation

To prepare for annotations, the mfMRI T2 map scans were exported from PACS into 3D Slicer (version 5.2.1) [18]. In 3D Slicer, the initial step was to take the first resting scan per participant as a reference and to annotate all muscles of interest (i.e., masseter, medial pterygoid, lateral pterygoid, intrinsic tongue, extrinsic tongue (genioglossus, hyoglossus, styloglossus, palatoglossus muscles), suprahyoid (digastric, geniohyoid, stylohyoid and mylohyoid), sternocleidomastoid, and the superior pharyngeal constrictor) using the Segment Editor Module. Because participants performed exercises outside the MRI, the additional scans were not in the exact same position. To optimize annotation efficiency and accuracy, the subsequent scans were registered on the reference scan using the Elastic module. The inverse transformation matrix was applied to visualize the initial annotations in their original spatial context. Any adjustments to these annotations were made only if they were shifted from the belly of the muscle. To ensure accurate annotations, researchers MN and MvA were trained by an experienced head and neck radiologist (LB), who also provided an interactive guide for assisting during annotations. MN and MvA annotated together in the same room, simultaneously. In case of disagreements, both researchers reviewed scans together to reach consensus. Subsequently, LB checked all annotations for correctness and accuracy. Once checked, T2 values (in ms) of each annotation were extracted.

### Statistical analysis

The primary study parameter was the change in muscle tissue T2 spin-spin relaxation time constant (ms) between pre- and post-exercise scans. Due to the small sample size (n = 5), only descriptive statistics were used. Median T2 map values for muscles of interest (pre- and post-exercise, left and right) were extracted, and the deltas (Δ) (post minus pre-exercise) were computed. Since the height of Δ is not directly proportional to the level of muscle activation, it cannot be used as a comparative measure, and we used a dichotomous activation indicator instead. For this, we determined whether the change in T2 map values exceeded the measurement noise. The Standard Error of the Median (SEM) was calculated using Median Absolute Deviations (MAD) and multiplied by 1.96, with which a 95% confidence interval around each individual measurement was calculated. T2 map values exceeding the upper limit of this interval were considered to indicate ‘true activation’ above noise. Bar charts showing the percentage true positive Δ T2 map values were created for each muscle group per exercise.

## Results

Every participant successfully underwent both pre- and post-exercise mfMRI. Four participants completed all six exercises, while one participant could not perform the Shaker exercise due to an uncontrollable cough reflex.

### Procedure

Although the study protocol included the exercises in a predetermined order, two participants started with the SEA exercises because they were unsure if they would be able to perform all exercises due to fatigue. Table 2. shows the sequence of exercises, the duration per exercise, the time of rest, and the duration to complete the study can be found. The median duration to complete the study was 119 min (range 91–144 min). The median duration of the conventional exercises was 9 min (range 6–11), with a rest period of 13 min (range 7–18). For the SEA exercises, the median exercise duration was 11 min (range 9–13), with a rest period of 11 min (range 4–17).

**Table 2.** Sequence and duration of exercises and pauses per participant in minutes

Sequence and duration in minutes of Exercises and Rest per participant													
		1		2		3	4			5		6	Total
P1	Exercise	cES	Rest	Masako	Rest	Shaker	Rest	CTAR	Rest	JOAR	Rest	ESAR	
	Duration	11	18	8	14	11	15	13	17	12	14	11	144
P2	Exercise	cES	Rest	Masako	Rest	Shaker	Rest	CTAR	Rest	JOAR	Rest	ESAR	
	Duration	8	12	6	11	6	16	9	16	10	15	11	120
P3	Exercise	CTAR	Rest	JOAR	Rest	ESAR	Rest	cES	Rest	Masako	Rest	Shaker	
	Duration	13	11	10	11	12	15	10	13	10	14	8	127
P4	Exercise	cES	Rest	Masako	Rest	Shaker	Rest	CTAR	Rest	JOAR	Rest	ESAR	
	Duration	11	10	11	10	9	11	10	10	10	4	9	105
P5	Exercise	CTAR	Rest	JOAR	Rest	ESAR	Rest	cES	Rest	Masako	Rest	Shaker	
	Duration	10	11	12	11	11	14	8	7	8	NA	NA	92
Summary per Exercises and Rest													
Exercise or rest Median (min-max)		cES 10 (7-11)	Rest 11 (7-18)	Masako 8 (6-11)	Rest 12 (10-14)	Shaker 9 (6-11)	Rest 15 (11-16)	CTAR 10 (9-13)	Rest 11 (10-17)	Rest 11 (10-17)	JOAR 10 (10-12)	Rest 11 (4-14)	ESAR 11 (9-12)
Summary per Exercise category					Conventional exercises			SEA exercises					
Median of Exercise duration (min-max)					9 (6-11)			11 (9-13)					
Median of Rest time duration (min-max)					13 (7-18)			11 (4-17)					

Abbreviations: Duration in minutes; *cES* conventional effortful swallow, *CTAR* Chin Tuck Against Resistance, *JOAR* Jaw Opening Against Resistance, *ESAR* Effortful Swallow Against Resistance, *SEA* Swallowing Exercise Aid

### Registration and annotation

**Fig. 2:**
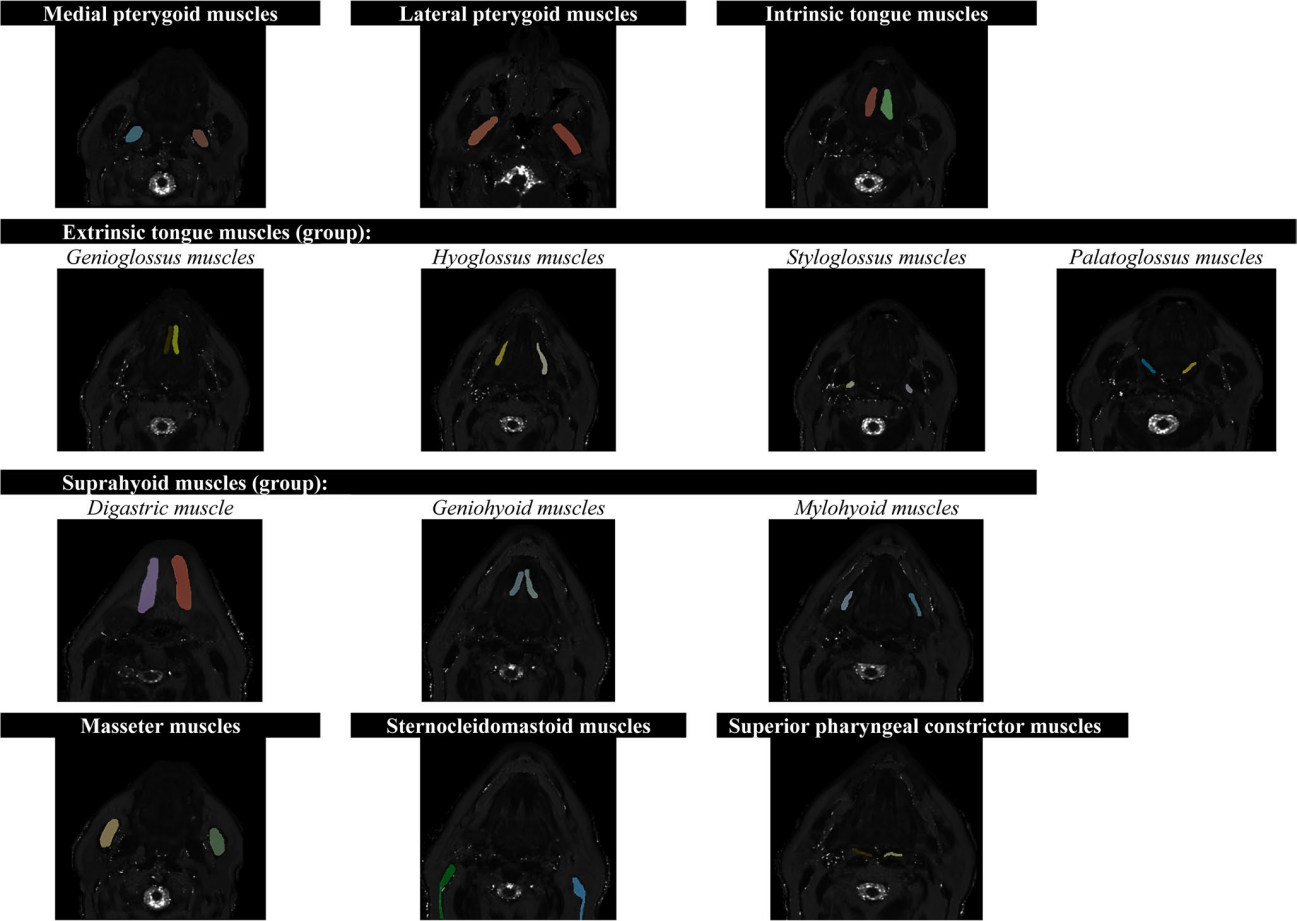
Overview of annotated muscles of interest in T2 map scans in laryngectomized participants

In total, 58 T2 map scans (29 pre-exercise and 29 post-exercise) were obtained from the five participants. Across these scans, fourteen muscles of interest bilaterally were annotated, resulting in 28 muscle annotations per scan and 1.624 muscle annotations in total. Out of these 1.624 annotations, the two annotators disagreed on131 muscles. Of these 131 muscles, 116 concerned the stylohyoid muscle, and the annotators asked LB for her opinion. LB checked all annotated muscles and excluded the stylohyoid muscle from the muscles of interest because it was not consistently localized, resulting in unreliable annotations and a lack of consensus. Eighteen annotations resulted in extreme T2 map values of “2047” for an unknown reason, which were considered errors and therefore excluded. These errors occurred in the masseter (6 annotations), sternocleidomastoid (2 annotations), and superior pharyngeal constrictor (10 annotations). The exclusion of the stylohyoid muscle and annotations with errors led to 1.490 muscle annotations available for analysis. In Fig. 2, an overview of the annotated muscles of interest is displayed.

### Muscle activation

**Fig. 3:**
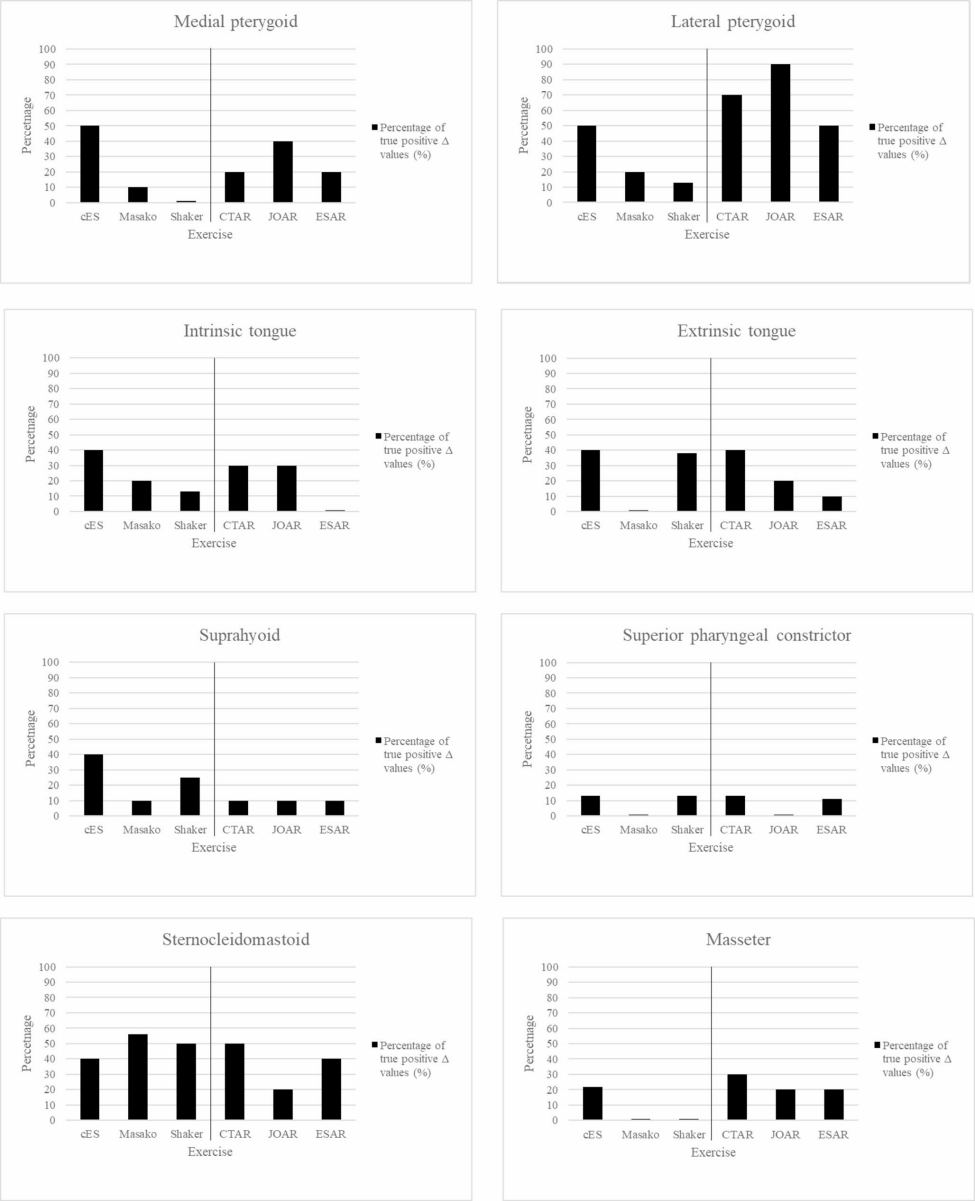
Bars of the percentage true (values exceeding standard error of the median*1.96) positive Δ T2 values in milliseconds for the muscles of interest after performing different swallowing exercises. Abbreviations: cES, conventional effortful swallowing; CTAR, Chin Tuck Against Resistance; JOAR, Jaw Opening Against Resistance; ESAR, Effortful Swallow Against Resistance; Midline, left: conventional exercises, right: resistance-based SEA exercisesbreviations:

In Tables 3. and 4, the Δ T2 map values for the eight muscles (groups), the SEM, and the percentages of true positive and negative Δ T2 values are displayed. In Fig. 3, bar charts of the percentages of true positive Δ T2 map values per muscle per exercise can be found.

**Table 3.** Δ T2 map values of muscles after performing conventional swallowing exercises per participant

Δ T2 map values per participant		P1		P2		P3		P4		P5		SEM		No. of true positive Δ values (%)		No. of true negative Δ values (%)	
		Left	Right	Left	Right	Left	Right	Left	Right	Left	Right	Left	Right				
*cES*	*Masseter*	−2	4	3	2	−6	NA	−14	−6	19	25	6	6	2/9	(22)	1/9	(22)
	*Lateral Pterygoid*	−7	−2	−1	−2	12	27	4	2	7	19	2	6	5/10	(50)	1/10	(10)
	*Medial Pterygoid*	4	1	−1	2	13	8	29	9	25	14	12	8	5/10	(50)	0/10	(0)
	*Intrinsic Tongue*	3	−6	−3	4	11	15	18	7	−1	2	6	4	4/10	(40)	1/10	(10)
	Extrinsic Tongue*	5	11	9	−4	3	0	29	23	16	2	10	10	4/10	(40)	0/10	(0)
	Suprahyoid**	2	4	10	13	−3	2	29	24	4	−4	4	6	4/10	(40)	0/10	(0)
	*SCM*	2	3	−2	3	60	95	79	0	7	35	8	8	4/10	(40)	0/10	(0)
	*SPC*	17	0	−24	−4	−6	−8	2	17	NA	NA	20	16	1/8	(13)	1/8	(13)
	% of activation													29/77	(38)	4/77	(5)
Masako	*Masseter*	−6	0	11	NA	2	NA	4	3	−3	3	14	4	0/8	(0)	0/8	(0)
	*Lateral Pterygoid*	1	7	1	1	−5	−9	1	−1	34	−18	2	4	2/10	(20)	3/10	(30)
	*Medial Pterygoid*	−4	−3	1	12	2	−6	4	2	−40	−5	8	10	1/10	(10)	1/10	(10)
	*Intrinsic Tongue*	−4	−25	2	7	3	−2	−3	4	−7	−1	2	6	2/10	(20)	4/10	(40)
	Extrinsic Tongue*	1	−6	3	6	2	4	−7	3	−11	2	6	16	0/10	(0)	2/10	(20)
	Suprahyoid**	−16	−10	7	20	−1	7	−11	−5	9	10	10	10	1/10	(10)	2/10	(20)
	*SCM*	42	−3	2	NA	12	88	93	22	0	2	6	10	5/9	(56)	0/9	(0)
	*SPC*	NA	NA	10	−2	9	6	−9	−6	NA	NA	14	14	0/6	(0)	0/6	(0)
	% of activation													11/73	(15)	12/73	(16)
Shaker	*Masseter*	3	5	−30	NA	NA	NA	8	6	NA	NA	14	6	0/5	(0)	1/5	(20)
	*Lateral Pterygoid*	−3	2	−2	−2	−9	4	11	−3	NA	NA	2	4	1/8	(13)	2/8	(25)
	*Medial Pterygoid*	−62	−65	−4	3	−8	9	−1	1	NA	NA	2	10	0/8	(0)	4/8	(50)
	*Intrinsic Tongue*	−26	−4	−2	0	−2	8	10	−14	NA	NA	8	10	1/8	(13)	2/8	(25)
	Extrinsic Tongue*	4	−9	5	5	11	7	10	25	NA	NA	6	14	3/8	(38)	0/8	(0)
	Suprahyoid**	−2	9	0	0	25	0	4	28	NA	NA	16	14	2/8	(25)	0/8	(0)
	*SCM*	5	68	47	NA	68	8	−48	3	NA	NA	16	4	4/8	(50)	1/8	(13)
	*SPC*	−48	NA	0	0	12	3	41	2	NA	NA	28	8	1/8	(13)	1/8	(13)
	% of activation													12/61	(20)	11/61	(18)

Abbreviations: Δ delta of T2 map value post-pre, P1-5 participant L, left, R right, *SEM* Standard Error of the Median multiplied by 1.96, *SCM* sternocleidomastoid muscle, *SPC* superior pharyngeal constrictor muscle, *cES* conventional effortful swallow exercise, *NA* not applicable, *Extrinsic tongue muscles included genioglossus, hyoglossus, styloglossus, and palatoglossus; **Suprahyoid muscles included digastric, geniohyoid, stylohyoid and mylohyoid; Bold = ‘true’value, exceeding the SEM

**Table 4:** Δ T2 map values of muscles after performing resistance-based swallowing exercises per participant

Δ T2 map values per participant		P1		P2		P3		P4		P5		SEM		No. of true positive Δ values (%)		No. of true negative Δ values (%)	
		Left	Right	Left	Right	Left	Right	Left	Right	Left	Right	Left	Right				
CTAR	*Masseter*	3	0	48	38	4	2	−6	−1	8	1	6	2	3/10	(30)	0/10	(0)
	*Lateral Pterygoid*	6	7	49	8	4	12	4	1	31	19	4	4	7/10	(70)	0/10	(0)
	*Medial Pterygoid*	−16	−7	6	27	−3	5	−15	−10	25	18	16	24	2/10	(20)	0/10	(0)
	*Intrinsic Tongue*	5	15	−18	7	1	4	23	−5	−15	3	6	4	3/10	(30)	3/10	(30)
	Extrinsic Tongue*	−17	−4	10	21	17	6	10	5	−8	6	6	8	4/10	(40)	2/10	(20)
	Suprahyoid**	15	−12	8	−2	9	7	−7	0	−2	9	10	10	1/10	(10)	1/10	(10)
	*SCM*	4	−2	87	97	5	6	58	−29	48	37	8	30	5/10	(50)	1/10	(10)
	*SPC*	−7	15	−8	35	NA	NA	−6	−6	−6	12	12	24	1/8	(13)	0/8	(0)
	% of activation													26/78	(33)	7/78	(9)
JOAR	*Masseter*	0	1	−10	−1	12	−1	4	−5	49	2	10	6	2/10	(20)	0/10	(0)
	*Lateral Pterygoid*	6	7	8	5	23	66	22	50	32	20	6	4	9/10	(90)	0/10	(0)
	*Medial Pterygoid*	−4	2	6	−5	17	32	−27	−71	39	12	14	4	4/10	(40)	3/10	(30)
	*Intrinsic Tongue*	8	0	1	5	14	−11	14	−36	4	4	8	4	3/10	(30)	2/10	(20)
	Extrinsic Tongue*	12	2	−5	−6	5	−1	16	−14	10	5	10	14	2/10	(20)	0/10	(0)
	Suprahyoid**	−4	−6	−37	−18	23	10	−4	0	−1	4	8	12	1/10	(10)	2/10	(20)
	*SCM*	−1	−3	70	−3	−40	99	11	−25	18	−7	28	30	2/10	(20)	1/10	(10)
	*SPC*	−18	5	1	−2	8	4	12	−30	−24	8	26	16	0/10	(0)	1/10	(10)
	% of activation													23/80	(29)	9/80	(11)
ESAR	*Masseter*	13	8	0	0	4	4	18	−8	−61	3	8	8	2/10	(20)	1/10	(10)
	*Lateral Pterygoid*	8	9	3	0	−6	−42	−11	70	3	−6	2	4	5/10	(50)	4/10	(40)
	*Medial Pterygoid*	42	3	1	8	10	−21	12	6	−50	0	16	6	2/10	(20)	2/10	(20)
	*Intrinsic Tongue*	0	5	5	−2	−7	−40	−1	−1	−21	−1	14	8	0/10	(0)	2/10	(20)
	Extrinsic Tongue*	−11	14	7	−3	−6	5	−13	2	−6	4	10	10	1/10	(10)	2/10	(20)
	Suprahyoid**	−6	6	0	−1	−1	1	−29	3	13	6	8	10	1/10	(10)	1/10	(10)
	*SCM*	45	55	−1	−2	8	8	55	44	−13	−22	22	22	4/10	(40)	0/10	(0)
	*SPC*	−5	5	4	1	20	4	15	5	−3	NA	16	20	1/9	(11)	0/9	(0)
	% of activation													15/79	(19)	12/79	(15)

Abbreviations: Δ delta of T2 map value post-pre, P1-5 participant, *L* left, *R* right, *SEM* Standard Error of the Median multiplied by 1.96, *SCM* sternocleidomastoid muscle, *SPC* superior pharyngeal constrictor muscle, *CTAR* Chin Tuck Against Resistance, *JOAR* Jaw Opening Against Resistance, *ESAR* Effortful Swallowing Against Resistance, *NA* not applicable, *Extrinsic tongue muscles included genioglossus, hyoglossus, styloglossus, and palatoglossus; **Suprahyoid muscles included digastric, geniohyoid, stylohyoid and mylohyoid; Bold = ‘true’ value, exceeding the SEM

**Fig. 4:**
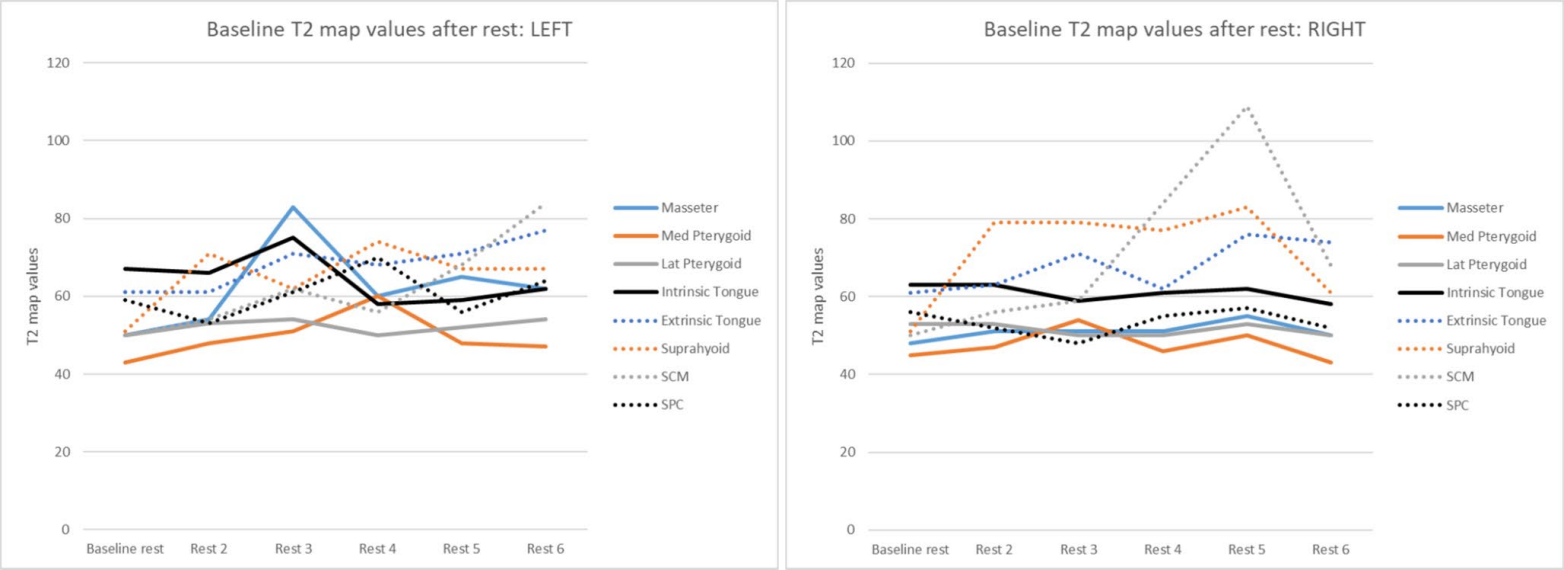
Baseline T2 map values after rest left and right

Overall, of the conventional exercises, the cES showed true positive Δ T2 map values in all eight muscles of interest, Masako in five, and Shaker in six. For the SEA exercises, true positive Δ T2 map values were found in all muscles of interest after performing CTAR, in seven after JOAR, and in six after ESAR. Among all annotated muscles, the percentage of true positive Δ T2 map values was highest for the lateral pterygoid muscles following the SEA exercises CTAR (70%) and JOAR (90%) (see Fig. 3). The Δ T2 map values were not consistently bilaterally activated (positive) or non-activated (negative) within the participants. Over the course of the exercise program, there was a trend towards (slightly) increased T2 map baseline values (see Fig. 4).

## Discussion

This study aimed to assess which muscles are activated after total laryngectomy with conventional swallowing exercises and swallow exercises performed against resistance with the SEA, using mfMRI based T2 maps. Results showed that in this patient population a range of swallowing muscles were activated with the applied conventional and resistance-based exercises, as indicated by true positive Δ T2 map values. Each of the six exercises activated at least three muscles of interest. Up to 90% of the muscles of interest (medial and lateral pterygoid, intrinsic and extrinsic tongue, suprahyoid, superior pharyngeal constrictor, masseter, and sternocleidomastoid muscles) showed true positive Δ T2 map values after performing the swallowing exercises, with the two highest percentages seen in the lateral pterygoids after performing the CTAR (70%) and JOAR (90%) exercises.

Dysphagia after laryngectomy has received little attention in the clinic and literature, partly because its symptoms differ from those in head and neck cancer patients with an intact larynx. Laryngectomized patients primarily experience issues with the efficiency of the (neo)pharyngeal phase and do not suffer from aspiration. Patients in the current study, the previously published clinical rehabilitation phase II trial with SEA, and a study on oral intake after laryngectomy all showed normal BMI values, which might hinder the timely recognition of dysphagia [10, 19]. 

In dysphagia rehabilitation various techniques are used. For immediate improvements in swallowing safety and efficiency, dietary modifications and compensatory techniques, such as changing body or head positions, are employed [20]. Additionally, specific swallowing exercises are applied to increase muscle strength and improve long-term swallowing physiology and bolus flow.

Swallowing with a larynx requires coordination among thirty different muscles in the head and neck [21]. After total laryngectomy, it is unclear which of these muscles, if still present, can be activated during swallowing exercises. We assumed that muscles not lying within the surgical area should keep their primary functions unaffected, such as the masseter and pterygoid muscles for jaw movements, and intrinsic and extrinsic tongue muscles for oral transport [22–24]. Results of the present study support this assumption, as these muscles showed the most extensive activation after all exercises.

We also assessed the muscles within the surgical area, suprahyoid muscles and superior pharyngeal constrictor, for their potential activation. The main function of the suprahyoid before surgery was to elevate the hyoid bone, but after laryngectomy, the hyoid bone is absent and these muscles are often repositioned, potentially affecting their activation. The superior pharyngeal constrictor before surgery had the function to contract the upper pharynx to help propel food downward into the esophagus [25, 26]. However, during the laryngectomy, the superior pharyngeal constrictor is detached from the thyroid cartilage and often sutured to the contralateral constrictor and to the suprahyoid muscles. Consequently, the activation and function of this muscle remain unknown. Nevertheless, the data show that despite all adaptations, some activation is still achievable following swallowing exercising.

Additionally, the sternocleidomastoid, which showed constant activation during spontaneous saliva swallowing according to literature, was included in the study [27]. In the current study, the sternocleidomastoid muscle showed activation after all swallowing exercises, even those in which the muscle would not be involved. This activation may have been caused by moving from supine position into sitting upright position to perform the exercises (and vice versa).

Swallowing exercises aiming to increase muscle strength, require training according to principles of muscle strength training, including progressive overload at 60–80% of one-repetition maximum (1-RM) or in elderly people also already achievable at 40–60% of the 1-RM [9]. Although conventional exercises lack this principle, this study found activation in all muscles of interest after the cES. The Masako exercise activated five muscles: medial and lateral pterygoid, intrinsic tongue, suprahyoid, and sternocleidomastoid. The Shaker exercise activated six muscles: lateral pterygoid, intrinsic and extrinsic tongue, suprahyoid, superior pharyngeal constrictor, and sternocleidomastoid.

The unique aspect of the SEA is that muscles can be trained with resistance optimized per patient and can be adjusted when the patient increases their strength. Performing resistance-based exercises with the SEA resulted in activation of all muscles after the CTAR exercise. The JOAR exercise activated the medial and lateral pterygoid, intrinsic and extrinsic tongue, suprahyoid, and sternocleidomastoid and masseter muscle. And the ESAR showed activation in the medial and lateral pterygoid, extrinsic tongue, suprahyoid, superior pharyngeal constrictor, sternocleidomastoid and masseter.

Although the current study explicitly focused on muscle activation after performing conventional and SEA exercises in laryngectomized patients, it did not investigate the addition of progressive load to the SEA exercises or determine which exercise type is most effective (conventional or resistance-based) in this patient population. However, the study found that both conventional and SEA exercises activated essential swallowing muscles, with SEA exercises activating more muscles of interest. Given that SEA aligns with muscle strengthening principles, it is likely that SEA training is more effective in increasing muscle strength. These findings support the results of a recently published clinical phase II trial, which showed that six weeks of SEA training improved muscle strength, swallowing capacity, and swallowing efficiency [10]. 

In our study, muscle activation symmetry varied across exercises. Only the lateral pterygoid muscle showed symmetric ΔT2 map values after CTAR and JOAR resistance-based SEA exercises, similar to healthy participants in our earlier study [11]. Other muscles often showed asymmetric activation, likely due to surgical impact and muscle reattachment. In our earlier study, some asymmetry was noted in healthy adults, as well, suggesting that natural variations may also explain these findings.

In the current study, the T2 map values differ from those reported in our earlier study [12], with the present values generally being somewhat higher. However, we have to stress once more that the height of Δ is not directly proportional to the level of muscle activation, so it cannot be used as a comparative effect measure. We hypothesized that these differences probably relate to the different study participants, because we used the same MRI, T2 map protocol, and head-spine coil. In our earlier study we included healthy young adults with an age range of 25–31 years, whereas the present study participants were older, with an age range of 54–67 years [12]. Further, although in the earlier study BMI was not mentioned, it is likely that in the present study all included participants had a higher BMI (all above 25). It is possible that this higher BMI has influenced the viscosity of muscle cells and therefore the observed T2 map values. Another aspect that might have had influence on the T2 map values is the fact that our participants underwent total laryngectomy and radiation therapy, resulting in scar tissue and loss of muscle volume in the targeted area [28, 29]. 

## Limitations

A key limitation of this study is the small sample size (*n* = 5) of laryngectomized individuals with a specific T/Y-shaped closure of the neopharynx, which restricts statistical power and may limit our ability to detect subtle effects of the swallowing exercises on muscle activation. Consequently, the findings may not be fully generalizable to the broader, more diverse population of total laryngectomy patients, given the heterogeneity in surgical techniques, surgical closures, patient characteristics, and rehabilitation outcomes.

Additionally, the MRI measurement method is not “fine-grained” with some uncertainties. For example, the long scanning time (4 min and 33 s) potentially resulted in loss of activation that was present during the exercise. Additionally, the voxel size during acquisition was 1.2 × 1.2 mm in-plane, with a reconstructed voxel size of 0.6 × 0.6 mm, using contiguous slices, each 3 mm thick. This could have resulted in the inclusion of other tissues alongside the annotated muscle, such as small blood vessels or fat.

Due to limited MRI space from the head-spine coil, participants performed swallowing exercises outside the MRI in an upright position, causing a 1-minute delay that might have affected T2 map or activation values. Fleckenstein et al. note that muscle rest can take 20–30 min; our maximum rest time was 18 min [30]. Despite the lymph massage protocol, baseline T2 map values increased slightly throughout the study. Extending rest periods would have made the study duration unfeasible. Future research should consider a 20–30-minute rest period between exercises.

All participants in this study were male, which may limit the generalizability of the findings to females. Anatomical and physiological differences between genders could affect muscle activation during swallowing exercises, highlighting the need for future research to include a more gender-diverse sample.

## Future research

The effectiveness of the conventional swallowing exercises in laryngectomized participants is currently unknown, but in view of the absence of adjustable and increasing resistance, similar improvements in muscle strengthening and swallowing function as seen with resistance-based exercises are unlikely. Nevertheless, it still would be interesting to carry out a randomized controlled trial with both conventional and resistance-based swallowing exercises included in the study protocol in this patient population.

## Conclusion

This study shows that after total laryngectomy muscles involved in swallowing can be activated with a range of swallowing exercises. Each exercise (conventional and with the SEA) activated at least three muscles of interest, with the number of activated muscles varying per exercise. The SEA exercises CTAR and JOAR led to the highest percentages of bilateral activation in the lateral pterygoid, but also in several other muscle groups.

## Supplementary Information

Below is the link to the electronic supplementary material.

Supplementary Material 1 (DOCX. 191 KB)

## Data Availability

The dataset generated and analyzed during the current study are available from the corresponding author upon reasonable request.
