# Supplementary material for: MfMRI assessment of muscle activation by swallowing exercises in laryngectomized individuals: investigating conventional and resistance-based exercises using the swallow exercise aid (SEA)
Source: Eur Arch Otorhinolaryngol. 2025 Nov 5;282(12):6519–32. doi: 10.1007/s00405-025-09727-9 (PMC12680836; doi:10.1007/s00405-025-09727-9)
Supplement: Supplementary file 1 — Supplementary Material 1 (DOCX. 191 KB) [file 405_2025_9727_MOESM1_ESM.docx]

# Appendix 1: Lymph edema massage protocol

**Purpose**
To activate and support lymphatic drainage in the head and neck area to bring the water ratio back to baseline in the activated muscles before proceeding to the next pre-exercise T2 map mfMRI scan.

**Material**

1. Foam Roller (max 5cm wide)
2. Clock, (Stop)watch or timer

**Protocol (min. 10 minutes)**

*Breathing exercises (min. 2 minutes)*

Let the participant…

- Sit comfortable in upright position.
- Place one hand on his/her chest and the other on the abdomen.
- Inhale slowly and deeply for a couple of seconds.
- Exhale slowly and completely for a couple of seconds.


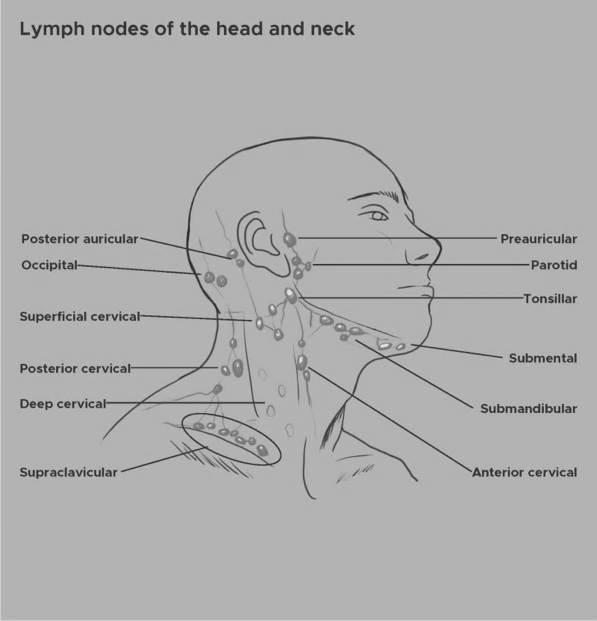
*Massage, performed by the SLP (min. 5 minutes)*

Roll in as straight a line as possible...

- From behind the ear to the clavicle.
- From below the ear to the clavicle.
- From submandibular to the clavicle.
- From in front of the ear to the jawline.
- From the cheekbone to the jawline.
- From the side of the nose to the jawline.

*Relax (min. 3 minutes)*

Inform the participant about the next exercise.

Figure 5 Lymph nodes of the head and neck, image published in Burkhead et al.

**Note**

1. In the case of a scar, you should follow the scar. Scar tissue can act as a barrier to the drainage of fluids.
2. Participants who have undergone RT may experience reduced drainage of lymphatic fluid due to fibrosis.

Neck lymph node dissection: Identify which lymph nodes are still present on forehand.
